# Effects of Dietary Alpha-Lipoic Acid on Growth Performance, Serum Biochemical Indexes, Liver Antioxidant Capacity and Transcriptome of Juvenile Hybrid Grouper (*Epinephelus fuscoguttatus*♀ × *Epinephelus polyphekadion*♂)

**DOI:** 10.3390/ani13050887

**Published:** 2023-02-28

**Authors:** Guanghai Ou, Ruitao Xie, Jiansheng Huang, Jianpeng Huang, Zhenwei Wen, Yu Li, Xintao Jiang, Qian Ma, Gang Chen

**Affiliations:** 1 Fishery College, Guangdong Ocean University, Zhanjiang 524088, China; 2 Guangdong Evergreen Feed Industry Co., Ltd., Zhanjiang 524000, China; 3 Guangdong Provincial Key Laboratory of Pathogenic Biology and Epidemiology for Aquatic Economic Animals, Zhanjiang 524088, China

**Keywords:** juvenile hybrid grouper, α-lipoic acid, growth performance, serum biochemical indexes, antioxidant capacity, transcriptome

## Abstract

**Simple Summary:**

As global demand for animal protein increases, the entire animal production system is gradually moving towards intensification. The aquaculture industry is growing rapidly, but it is vulnerable to disease and environmental stress, resulting in aquaculture losses. Antioxidant supplementation in diets can improve the resistance of fish to environmental stress, which is an important measure to reduce the loss of the aquaculture industry. Alpha-lipoic acid (α-LA) is considered to be a “general antioxidant” or “ideal antioxidant” which has a strong antioxidant capacity. In this study, juvenile hybrid groupers were fed a diet supplemented with α-LA for 56 days. The results indicated that the addition of 0.4 and 0.6 g/kg α-LA to the diet inhibited the growth performance of juvenile hybrid groupers. Furthermore, 1.2 g/kg α-LA could reduce the blood lipid level, improve hepatocyte damage, and increase the antioxidant enzyme activity of the liver. In addition, transcriptome results indicated that dietary α-LA significantly affected the pathway related to immune function (the JAK/STAT signaling pathway, prolactin signaling pathway, and antigen processing and presentation) and glucose homeostasis (glycolysis/gluconeogenesis).

**Abstract:**

We aimed to investigate the effects of dietary alpha-lipoic acid (α-LA) on the growth performance, serum biochemical indexes, liver morphology, antioxidant capacity, and transcriptome of juvenile hybrid groupers (*Epinephelus fuscoguttatus*♀ × *Epinephelus polyphekadion*♂). Four experimental diets supplemented with 0 (SL0), 0.4 (L1), 0.6 (L2), and 1.2 (L3) g/kg α-LA were formulated and fed to three replicates of juvenile hybrid grouper (24.06 ± 0.15 g) for 56 d. The results indicated that dietary 0.4 and 0.6 g/kg α-LA significantly decreased the weight gain rate in juvenile hybrid groupers. Compared with SL0, the content of total protein in the serum of L1, L2, and L3 increased significantly, and alanine aminotransferase decreased significantly. The content of albumin in the serum of L3 increased significantly, and triglyceride, total cholesterol, and aspartate aminotransferase decreased significantly. In addition, the hepatocyte morphology in L1, L2, and L3 all showed varying degrees of improvement, and the activities of glutathione peroxidase and superoxide dismutase in the liver of L2 and L3 were significantly increased. A total of 42 differentially expressed genes were screened in the transcriptome data. KEGG showed that a total of 12 pathways were significantly enriched, including the pathway related to immune function and glucose homeostasis. The expression of genes (*ifnk*, *prl4a1*, *prl3b1*, and *ctsl*) related to immune were significantly up-regulated, and the expressions of *gapdh* and *eno1* genes related to glucose homeostasis were significantly down-regulated and up-regulated, respectively. In summary, dietary supplementation of 0.4 and 0.6 g/kg α-LA inhibited the growth performance of juvenile hybrid groupers. A total of 1.2 g/kg α-LA could reduce the blood lipid level, improve hepatocyte damage, and increase the hepatic antioxidant enzyme activity. Dietary α-LA significantly affected the pathway related to immune function and glucose homeostasis.

## 1. Introduction

As global demand for animal protein increases, the entire animal production system is gradually moving towards intensification [1]. The aquaculture industry is growing rapidly, but it is vulnerable to the interactions between the animals themselves, diseases, and the environment. Therefore, a new model of aquaculture management strategy has emerged as a result of a growing understanding of animal nutrition and feed. The core objectives of this model are to minimize the effects of stressors by neutralizing free radicals, repairing oxidative damage to biological macromolecules and membrane systems, enhancing immunity, and maintaining normal physiological homeostasis. The key points of this model are antioxidant supplementation and increasing endogenous cellular antioxidants [1]. Supplementation of diets with antioxidants can improve the resistance of fish to environmental stresses and is an essential measure to reduce losses in the aquaculture industry [2]. Alpha-lipoic acid (α-LA), also known as 1,2-dithiolane-3-valeric acid, with the molecular formula C_8_H_14_O_2_S_2_, was first isolated from pig liver by Lester J. Reed in 1951 [3]. α-LA is a naturally occurring compound found in microorganisms, plants, and animals, and is considered to be an “ideal antioxidant” or “universal antioxidant” because of its strong antioxidant capacity [4,5].

Studies have shown that α-LA can improve the survival rate, growth performance, and immunity of fish, and also improve the nutritional value of fish, which makes α-LA suitable for application in aquaculture [6]. For example, dietary supplementation with an appropriate amount of α-LA could promote growth, fatty acid β-oxidation, and lipolysis of grass carp (*Ctenopharyngodon idellus*; Cuvier et Valenciennes, 1844), increase protein deposition, enhance immunity and antioxidant capacity, alleviate the inflammatory response, and reduce lipid oxidative damage. It also could promote the expression of peripheral anorexia factor mRNA and reduce the expression of peripheral appetite factor mRNA, thus, reducing the intake and body weight of grass carp [7,8,9,10]. The enhancement of growth performance has also been found in other aquatic organisms with moderate amounts of α-LA in their diets, such as Nile tilapia (*Oreochromis niloticus*; Linnaeus, 1758) [11], African catfish (*Clarias gariepinus*; Burchell, 1822) [2], giant gourami (*Osphronemus goramy*; Lacepède, 1801) [12], Chinese mitten crab (*Eriocheir sinensis*; H. Milne Edwards, 1853) [13], and northern snakehead (*Channa argus*; Cantor, 1842) [14]. In addition, dietary supplementation with moderate amounts of α-LA could promote the expression of gluconeogenesis-related genes induced by a high-fat diet in fish, reduce lipid accumulation under high-fat conditions [15], and enhance starch utilization in carp (*Cyprinus carpio*; Linnaeus, 1758) [16].

The hybrid grouper (*Epinephelus fuscoguttatus*♀; Forsskål, 1775 × *Epinephelus polyphekadion*♂; Bleeker, 1849) is an important mariculture fish in southern China, with the characteristics of rapid growth and strong stress resistance, and it has a high economic value in China [17,18,19,20]. Although α-LA has been studied for over 70 years and there have been numerous studies on its addition as an antioxidant of aquatic animal diets, there have been few studies of α-LA in terms of the supplementation of marine fish diets. There are no reports of α-LA being added to the diet of groupers. Therefore, the purpose of this experiment was to research the effects of diet supplementation with α-LA on the growth performance, serum biochemical indexes, hepatic morphology, antioxidant capacity, and transcriptome of juvenile hybrid grouper fish, and to expand theoretical knowledge for the application of antioxidants in the hybrid grouper diet.

## 2. Material and Methods

### 2.1. Preparation of Diets and Testing of Nutritional Levels

Four isonitrogenous diets (SL0, L1, L2, and L3) were prepared with 0, 0.4, 0.6, and 1.2 g/kg of α-LA (99% purity, Yingbo biotechnology Co., Ltd.), respectively. The α-LA content was referenced from previous studies [2,7,12]. Referring to the study on experimental diet formulation and nutrient levels of hybrid groupers by Xie et al. [20]. The experimental diet formulations, as well as nutrient levels, are shown in Table 1. All feed raw materials were crushed and sieved through a 60-mesh sieve. We weighed the ingredients accurately according to the feed formula and mixed them well, then added the fish oil and soybean lecithin, rubbed the powdered ingredients and oil together manually, then added the right amount of water to knead all the ingredients into a dough. Finally, the raw material was processed into pellets with a particle size of 2.5 mm using a twin-screw extruder, air-dried, and stored in a −20 °C refrigerator. The nutritional levels of the diets were tested according to the AOAC standard method [21], and specific detection methods refer to An et al. [22].

### 2.2. Experiment Design

The experiments were conducted in the Zhanjiang Marine Hi-tech Park of Guangdong Ocean University (Zhanjiang, China). The water used for the culture experiments was natural seawater treated by sand filtration and sedimentation with uninterrupted aeration. The water temperature was kept at 28.5 ± 2.0 °C, pH was maintained at 7.6–8.2, dissolved oxygen was kept above 6 mg/L, total nitrite and ammonia content was kept below 0.04 mg/L, and the photoperiod adopted a natural day–night cycle (12 h of light, 12 h of darkness). The experimental juvenile hybrid groupers were purchased from a grouper hatchery at the southeast quay of Zhanjiang City, Guangdong Province, China, and were temporarily reared in an indoor cement pond (2.0 m × 4.0 m × 2.0 m) for 14 days after being transported back to the base. At the end of temporary rearing, the fish were starved for 24 h. A total of 360 fish (24.06 ± 0.15 g) with similar sizes, intact scales, and normal diet were randomly allotted to 12 fiberglass tanks (0.5 m^3^). Twelve fiberglass tanks were divided into four groups (SL0, L1, L2, and L3) with three replicates per group. The experiment was carried out in an indoor flowing water aquaculture system for 56 days. During the experiment, the corresponding feed was fed at 8: 30 and 17: 00 every day at 5–8% of their body weight. The water was changed as necessary to maintain superior water quality.

### 2.3. Sample Collection

After the end of the experiment, all the experimental fish were fished out and weighed after 24 h starvation treatment. Six fish were randomly fished out from each fiberglass tanks and anesthetized with eugenol (each 100 mg of eugenol is dissolved in 1 L of seawater, Shanghai Yuanye Biotechnology Co., Ltd., Shanghai, China). The body length and body weight were measured to calculate morphological indices. Subsequently, blood was collected from the tail vein of the fish using a 1 mL needle tube, and the needle tube was washed with heparin sodium before blood collection. After standing for 12 h at 4 °C, the blood was centrifuged using a refrigerated high-speed centrifuge (4 °C, 3500 rpm,10 min). The supernatant (serum) was collected and placed in a 2 mL centrifuge tube and stored at −80 °C for biochemical testing. The abdomen was dissected using sterilized scissors and tweezers, the visceral mass and liver were separated and weighed, and the liver was then washed with saline to remove other impurities. A liver tissue (5 mm × 5 mm × 5 mm) was cut from the center of the liver and placed in a 2 mL centrifuge tube containing 4% paraformaldehyde. After 24 h of fixation, the liver tissue was washed with 70% ethanol solution and stored in 70% ethanol solution for HE sections. About 1.0 g of liver tissue was cut from the remaining liver tissue and placed in a 2 mL cryopreservation tube, frozen in liquid nitrogen, and transferred to a −80 °C refrigerator for antioxidant capacity testing and transcriptome sequencing.

### 2.4. Growth Performance Index Measurement

In this study, the calculation methods of the growth performance indexes of juvenile hybrid groupers are as follows:

(1) Weight gain rate (WGR,%)=[final body weight (g) − initial body weight (g)] / initial body weight (g)×100%;

(2) Survival rate (SR, %)= The number of final fish / The number of initial fish×100;

(3) Feed conversion ratio (FCR)= feed intake (g) / [final body weight (g) − initial body weight (g)];

(4) Specific growth rate (SGR,%/d)={Ln[final body weight (g)] − Ln[initial body weight (g)]} / Number of days (d)×100%;

(5) $$\mathrm{Condition}\;\mathrm{factor}\;\left(\mathrm{CF},\;g/{\mathrm{cm}}^{3}\right)=\left[\mathrm{final}\;\mathrm{body}\;\mathrm{weight}\;\left(g\right)\;/\;\mathrm{Final}\;\mathrm{body}\;\mathrm{length}\;{\left(\mathrm{cm}\right)}^{3}\right]\times 100\%;$$

(6) $$\mathrm{Feed}\;\mathrm{intake}\;\left(\mathrm{FI},\%\;\right)=\frac{\mathrm{total}\;\mathrm{feed}\;\mathrm{weight}\;\left(g\right)}{\left\{\left[\mathrm{initial}\;\mathrm{body}\;\mathrm{weight}\;\left(g\right)+\mathrm{final}\;\mathrm{body}\;\mathrm{weight}\;\left(g\right)\right]/2\right\}\times \mathrm{Number}\;\mathrm{of}\;\mathrm{days}\;\left(d\right)}\times 100$$

(7) Hepatosomatic index (HSI, %)=[liver weight (g)/ weight of this fish (g)]×100%;

(8) Viscerosomatic index (VSI, %)=[viscera weight (g)/ weight of this fish (g)]×100%

### 2.5. Determination of Serum Biochemical Indexes and Liver Antioxidant Parameters

The serum biochemical indexes included triglyceride (TG, GPO-PAP enzymatic method), total cholesterol (TCHO, COD-PAP method), total protein (TP, BCA microplate method), albumin (ALB, bromocresol green method), low-density lipoprotein cholesterol (LDL-C, dual reagent direct method), high-density lipoprotein cholesterol (HDL-C, dual reagent direct method), aspartate aminotransferase (AST, Lai’s method), and alanine aminotransferase (ALT, Lai’s method). Antioxidant parameters of the liver included glutathione peroxidase (GSH-Px, colorimetric method), catalase (CAT, ammonium molybdate method), superoxide dismutase (SOD, WST-1 method), and malondialdehyde (MDA, TBA method). The above indicators were determined using kits produced by Nanjing Jiancheng Bioengineering Institute (Jiangsu, China). Experiments were conducted in strict accordance with the instructions, and all instructions can be found and downloaded at http://www.njjcbio.com (accessed on 15 January 2023).

### 2.6. Preparation and Observation of Hematoxylin-Eosin Stained Liver Sections

The fixed liver tissue was taken out and repaired with a scalpel and placed in a dehydration box. The liver tissue was dehydrated by gradient alcohol using a dehydrator (Donatello, DIAPATH) and then embedded in paraffin. The paraffinized liver tissue was embedded in an embedding machine (JB-P5, Wuhan Junjie Electronics Co., Ltd., Wuhan, China) to form a tissue block. After cooling, 4 μm thick sections were cut out in a paraffin sectioning machine (RM2016, Shanghai Leica Instrument Co., Ltd., Shanghai, China). After deparaffinization, the sections were stained with hematoxylin and eosin, and finally dehydrated and fixed in a glass slide. The morphology of liver cells was observed using an upright fluorescence microscope (Nikon ECLIPSE Ni-E, Tokyo, Japan).

### 2.7. Transcriptome Sequencing and Analysis

#### 2.7.1. RNA Extraction and Detection, Library Construction and High-Throughput Sequencing

Total RNA from SL0 and L3 hepatic tissues was extracted using a Trizol kit (Invitrogen, Carlsbad, CA, USA). The total RNA quality and integrity were examined using an Agilent 2100 biological analyzer (Agilent Technologies, Palo Alto, CA, USA) and RNase-free agarose gel electrophoresis, respectively. The library construction and high-throughput sequencing were completed by Genedenovo Biotechnology Co., Ltd. (Guangzhou, China), and high-throughput sequencing was performed using an Illumina NovaSeq 6000.

#### 2.7.2. Data Quality Control, De Novo Assembly and Unigene Basic Annotation

The raw data were quality-controlled using the quality control software fastp (version 0.18.0) to filter low-quality raw sequencing data. De novo assembly was performed using the short reads assembling the program, Trinity. The unigene sequence was compared with the SWISS-PROT protein database, NCBI non-redundant protein (Nr) database, Kyoto Encyclopedia of Genes and Genome (KEGG) database, and COG/KOG database using the BLASTx program, and then the protein function annotation was obtained according to the best alignment results.

#### 2.7.3. Differentially Expressed Genes Analysis

The analysis was performed using DESeq2 software. First, we normalized the read counts, then calculated the probability of hypothesis testing (*p*-value) based on the model, and, finally, we performed multiple hypotheses testing corrections to obtain the FDR value (false discovery rate). Based on the results of differential analysis, the genes of FDR < 0.05 and |log2(Fold Change)| > 1 were screened as differentially expressed genes (DEGs). Volcano plot analysis, KEGG pathway enrichment analysis, and GO functional enrichment analysis were performed according to the DEGs.

#### 2.7.4. Real-Time Quantitative PCR (RT-qPCR) Validation

Five genes, namely phosphatase inhibitor-1 (*i-1*), alpha-enolase (*eno1*), thioredoxin-interacting protein (*txnip*), parvalbumin (*pvalb*), and dual specificity phosphatase 1 (*dusp1*), were randomly selected from DEGs for RT-qPCR to verify the reliability of RNA-Seq data. Total RNA extraction, cDNA synthesis, and RT-qPCR detection of SL0 and L3 liver tissues were performed using TransGen Biotech kits (Beijing, China). The specific primers (Table 2) were designed by Primer Premier 5.0 software and was synthesized by Sangon Biotech Co., Ltd. (Shanghai, China). According to the RT-qPCR kit instructions, qualified cDNA and primers were tested using a LightCycler 96 real-time fluorescent quantitative PCR instrument (Roche, Basel, Switzerland) with four replicates for each sample. The reaction procedures were as follows: 94 °C for 30 s (1 cycle); 95 °C for 5 s, 60 °C for 15 s and 72 °C for 10 s (40 cycles); 95 °C for 10 s, 60 °C for 60 s and 95 °C for 1 s (1 cycle); 37 °C for 30 s (1 cycle). According to the measured Ct value, the relative expression of each gene was calculated using the 2^−ΔΔCt^ method [23].

### 2.8. Data Statistical Analysis

One-way ANOVA was performed on the experimental data using SSPS 21.0. For WGR, SR, SGR, VSI, and HSI, data transformation was required to remove the % before performing ANOVA, and, after completing ANOVA, data transformation was performed and the % was added. Tukey’s test was used for multiple comparisons if there were significant differences between groups. The experimental data were expressed as mean ± standard deviation (mean ± SD). Here, *p* < 0.05 represents a significant difference between groups.

## 3. Results

### 3.1. Growth Performance

The effects of dietary α-LA supplementation on the growth performance, morphology, and feed utilization of juvenile hybrid groupers are shown in Table 3. The WGR was significantly lower in the L1 and L2 groups than in SL0 and L3, and the FCR was significantly higher in the L2 group than in the other groups (*p* < 0.05). There was no significant difference in other indicators.

### 3.2. Serum Biochemical Indexes

The effects of dietary α-LA supplementation on the serum biochemical indexes of juvenile hybrid groupers are shown in Table 4. The TG level of L3 was significantly lower than that of SL0 and L1, and the TCHO level of L3 was significantly lower than that of SL0 (*p* < 0.05). The TP content of SL0 was significantly lower than the other three groups (*p* < 0.05). The ALB level of L3 was significantly higher than that of SL0 and L1 (*p* < 0.05). The LDL-C level of L2 was significantly higher than the other three groups, and the LDL-C level of L3 was also significantly higher than SL0 and L1 (*p* < 0.05). The AST level of L3 was significantly lower than the other three groups, while the ALT level of SL0 was significantly higher than the other three groups (*p* < 0.05).

### 3.3. Hepatic Morphology

**Figure 1:**
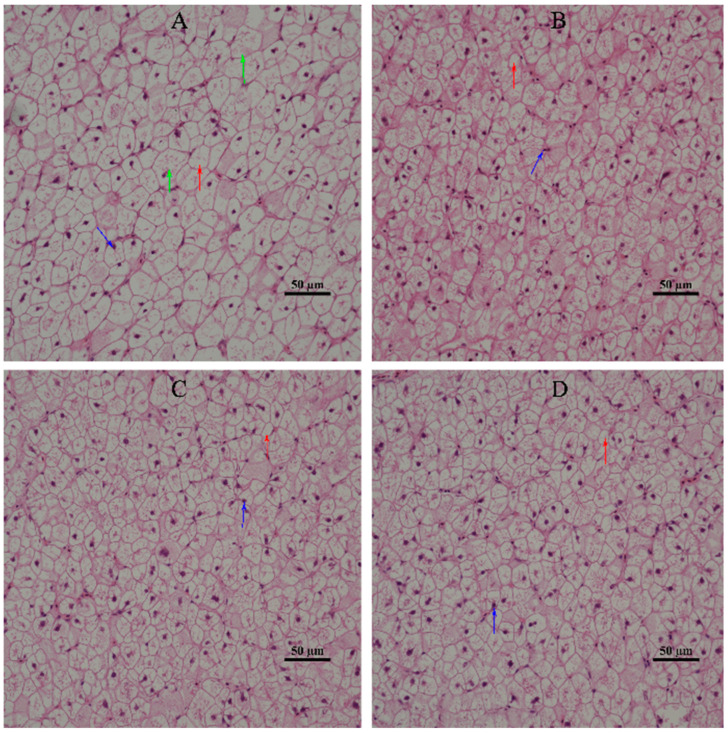
Effect of diet supplementation with α-LA on the hepatic histomorphology of juvenile hybrid groupers. The labels in the upper right corner represent the different experimental diets. (**A**) SL0; (**B**) L1; (**C**) L2; (**D**) L3. The red arrow represents cell vacuolation, the blue arrow represents cell nuclear migration, and the green arrow represents cell swelling. Scale bars = 50 µm; 400× magnification.

The effect of dietary α-LA supplementation on the hepatic morphology of juvenile hybrid groupers is shown in Figure 1. It was observed that the hepatic cells of the SL0 (control) group showed serious cell vacuolation, swelling, disordered arrangement, and nuclear migration. Compared with SL0, L1, L2, and L3 liver cells were slightly vacuolated, the phenomenon of nuclear migration was reduced, and cell morphology was more regular.

### 3.4. Antioxidant Capacity of Liver

The effect of dietary α-LA supplementation on the hepatic antioxidant capacity of juvenile hybrid groupers is shown in Table 5. The GSH-Px activity of L3 was significantly higher than that of the other three groups, and the GSH-Px activity of L1 and L2 was also significantly higher than that of SL0 (*p* < 0.05). The activity of SOD in L2 and L3 was significantly higher than that in SL0 and L1 (*p* < 0.05). There was no significant difference in CAT activity and MDA content (*p* > 0.05).

### 3.5. Liver Transcriptome

#### 3.5.1. Transcriptome Sequencing Results

The transcriptome data are shown in Table 6. A total of 37760784900 bp of RawData was obtained. After data quality control and filtering low-quality data, a total of 37,229,629,218 bp of CleanData was obtained. Base quality and composition analysis showed that the GC content range in each liver tissue sample was 49.58–50.10%, the percentage of Q20 bases was higher than 98.13%, and the percentage of Q30 bases was higher than 94.62%.

#### 3.5.2. DEGs Analysis

**Figure 2:**
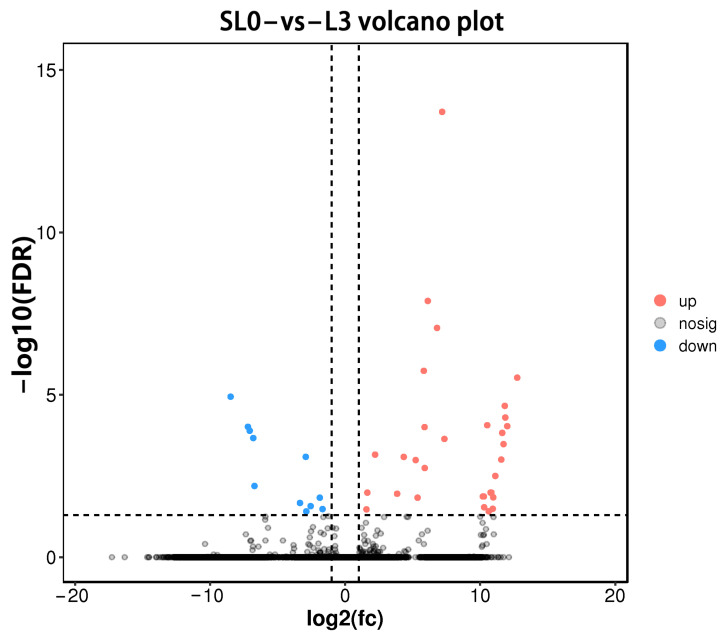
Volcano map of DEGs (SL0 vs L3). Red dots represent up-regulated genes, blue dots represent down-regulated genes, and grey dots represent genes with no significantly difference.

A total of 42 DEGs were identified using DEseq2 software under FDR < 0.05 and |log2(Fold Change)| > 1. Compared with the SL0, 31 DEGs were up-regulated and 11 DEGs were down-regulated in L3 liver tissue (Figure 2). A part of the DEGs is shown in Table 7.

#### 3.5.3. GO Function Analysis of DEGs

**Figure 3:**
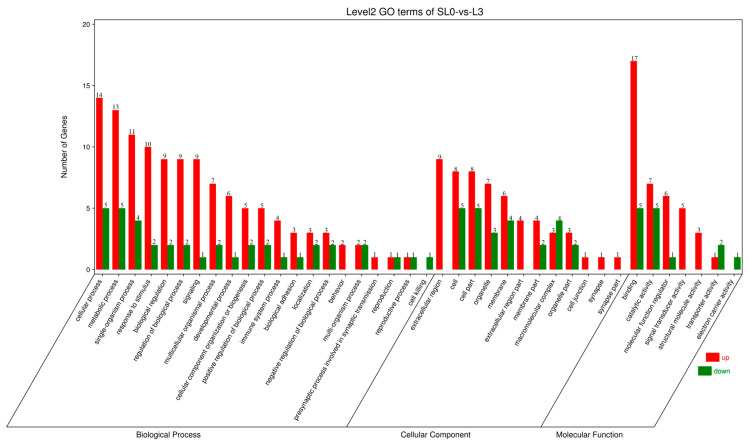
GO enrichment analysis of DEGs in the livers of juvenile hybrid groupers (SL0 vs L3). The abscissa is the enriched gene ontology (GO) term, and the ordinate is the number of differentially expressed genes in the term. The red columns represent up-regulated DEGs, and the green columns represent down-regulated DEGs.

GO functional enrichment analysis was performed on DEGs. Based on sequence homology, all DEGs were classified into the following three major branches of GO: molecular function, biological process, and cellular component, including 40 functional subcategories, involving 7 molecular functions, 12 cellular components, and 21 biological processes (Figure 3). Among them, the biological process is mainly composed of the single organism process, metabolic process, and cellular process. Cellular components were mainly the membrane, organelle, cell part, and cell. The main molecular functions were molecular function regulation, catalytic activity, and binding.

#### 3.5.4. KEGG Pathway Enrichment Analysis of DEGs

**Figure 4:**
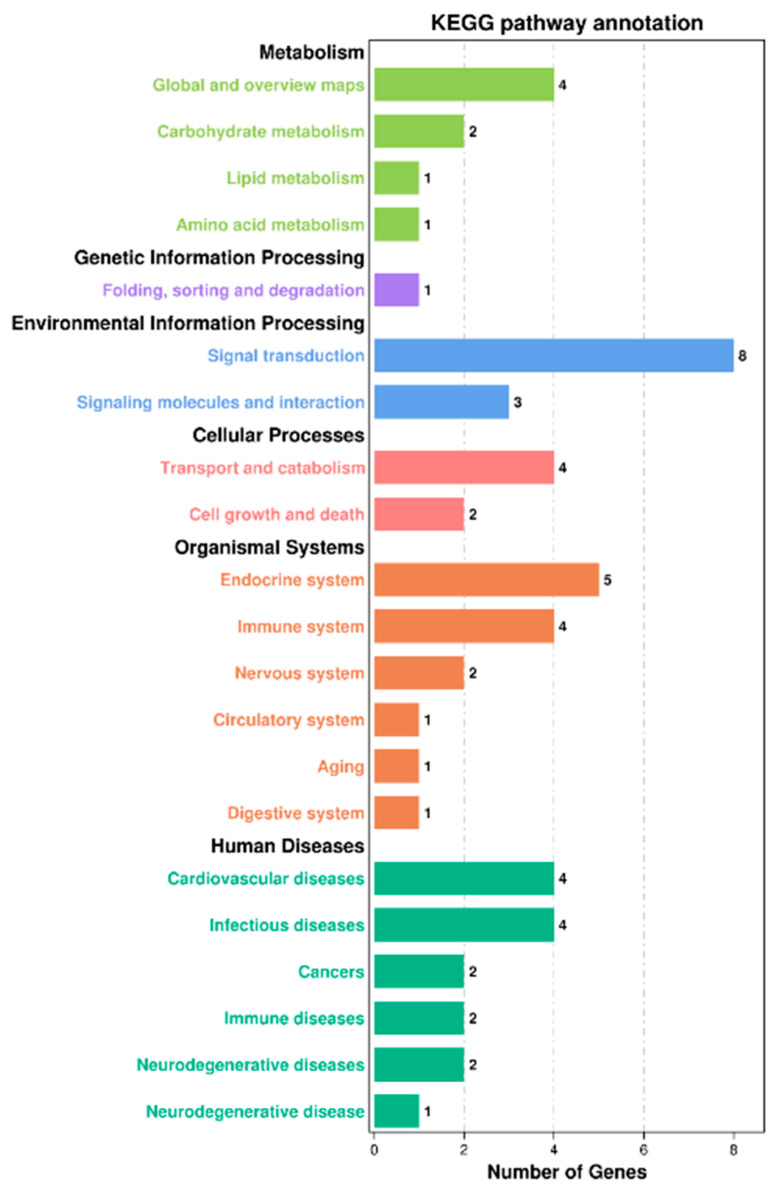
KEGG pathway enrichment analysis of DEGs in the livers of juvenile hybrid groupers (SL0 vs L3). The ordinate is the enriched KEGG subclass, and the abscissa is the number of genes enriched in this subclass. The number on each KEGG subclass item represents the number of differentially expressed genes on that item. Different colors represent different categories.

In the KEGG pathway database, biological metabolic pathways are divided into six categories, namely human diseases, organismal systems, cellular processes, environmental information processing, genetic information processing, and metabolism. In this experiment, a total of 17 DEGs were annotated into these six categories. DEGs were mostly enriched in the two KEGG main classes of biological systems and human diseases; they were also enriched in the overall and overview maps, signal transduction, endocrine system, immune system, cardiovascular disease, and infectious disease KEGG subclasses (Figure 4).

**Figure 5:**
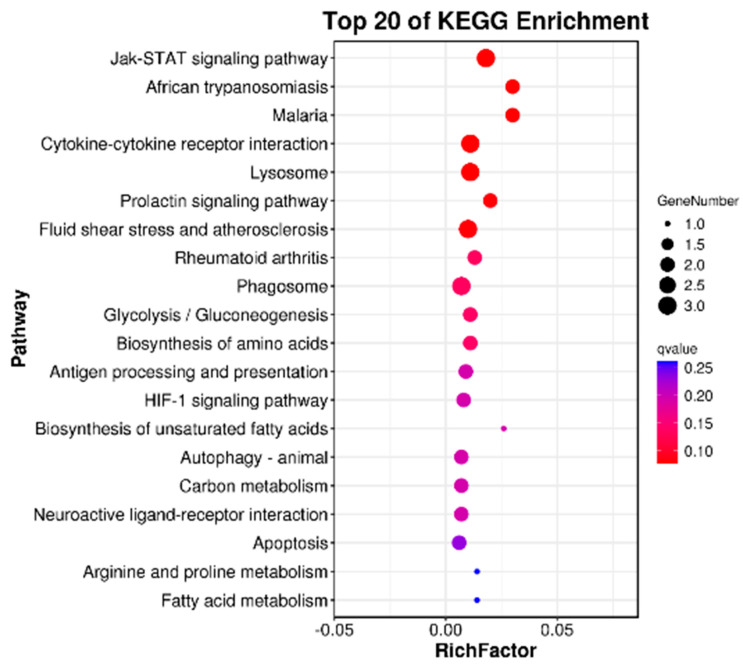
Top 20 KEGG pathways with significant enrichment of DEGs in the livers of juvenile hybrid groupers (SL0 vs L3). Ordinate represents the name of the pathway. The abscissa represents the enrichment factor, and the circle’s color represents Q. The deeper the red color, the more reliable the significant enrichment, and the larger the circle, the greater the number of enriched genes.

When performing KEGG pathway enrichment analysis on DEGs, the top 20 pathways with the smallest *p*-value were used to make KEGG enrichment bubble maps, and the results were shown in Figure 5. DEGs are significantly enriched in the JAK/STAT signaling pathway, glycolysis/gluconeogenesis, amino acid biosynthesis pathways, cytokine–cytokine receptor interaction, lysosomes, and so on. However, each KEGG significantly enriched pathway contained no more than three DEGs.

#### 3.5.5. Validation of RNA Sequencing Data

**Figure 6:**
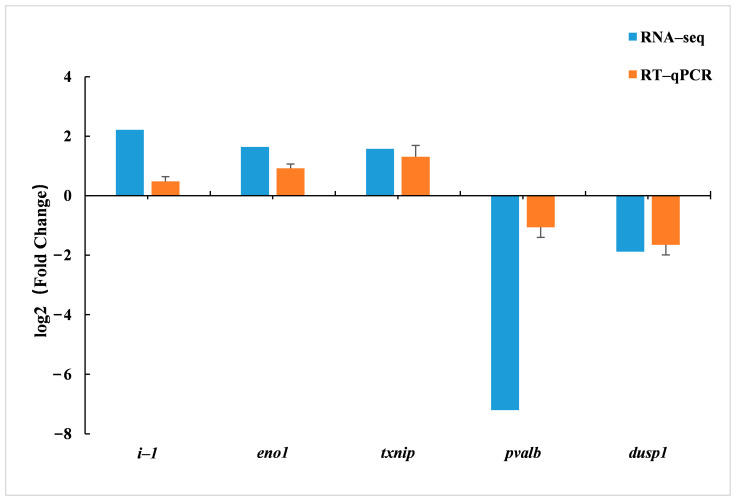
RT-qPCR verification of DEGs in the livers of juvenile hybrid groupers (SL0 vs L3).

To verify the accuracy of RNA-Seq results, five DEGs (two down-regulated and three up-regulated genes) were randomly selected, and their expression levels were detected by RT-qPCR. The results are shown in Figure 6. The results of the gene expression obtained were consistent with the trend of the results obtained from RNA-Seq, indicating that the RNA-Seq data had a certain feasibility.

## 4. Discussion

α-LA is a multifunctional antioxidant that can promote growth performance as a feed additive for poultry animals [24]. However, α-LA could also inhibit AMPKα in the hypothalamuses of chickens (*Gallus*; Linnaeus, 1758) to reduce food intake [25], and could activate AMPKα in the liver to inhibit the synthesis of glycogen synthase in the liver, resulting in a decrease in glycogen synthesis, thereby changing energy homeostasis and delaying the growth of chicken weight [26]. Therefore, the growth-promoting effect of α-LA needs to be analyzed specifically in combination with the amount of α-LA added. In the study of α-LA as a diet supplement for aquatic animals, more studies have shown that with the increase in α-LA dose, the growth performance of aquatic animals exhibited a trend of increasing first and then decreasing, and high doses of α-LA were still able to improve the growth performance, such as in catfish [2], giant gourami [12], northern snakehead [14], and Chinese mitten crab [27]. However, some studies have suggested that high doses of α-LA had an inhibitory effect on the growth of aquatic animals, such as in Nile tilapia [11] and oriental river prawn (*Macrobrachium nipponense*; de Haan, 1849) [28]. The recommended addition amounts were 439–528 mg/kg and 1354.8 mg/kg, respectively, but their growth performance was inhibited at 2400 mg/kg and 4000 mg/kg, respectively. In the present study, dietary supplementation with low doses of α-LA (0.4 and 0.6 g/kg) significantly reduced the WGR of juvenile hybrid groupers. Similar to the experimental results of Zhang et al. [29], the addition of lower α-LA to the diet reduced the WGR of abalone (*Haliotis discus hannai*; Ino, 1952), which may be the result of α-LA increasing energy consumption in juvenile hybrid groupers [30]. However, the addition of 1.2 g/kg α-LA had no significant effect on the WGR of juvenile hybrid groupers, which may be the result of a high dose of α-LA promoting lipolysis to consume energy by activating the AMPKα-ATGL pathway without causing weight loss [9]. In addition, Ding et al. [31] found that α-LA in diets could reduce the growth rate of oriental river prawn fed with low carbohydrate diet but had no significant effect on the growth rate of oriental river prawn fed with high carbohydrate diet. This indicates that the composition of the diet may affect the mechanism of α-LA. Huang et al. [7] discovered that dietary supplementation of 1.2 g/kg α-LA could inhibit the growth performance of grass carp. In this experiment, dietary supplementation of 1.2 g/kg α-LA had no significant effect on the WGR of juvenile hybrid groupers, indicating that different species had different sensitivities to α-LA. At present, the optimal α-LA addition amount for juvenile hybrid groupers with WGR as a reference still needs further study, and 1.2 g/kg α-LA has a certain reference value.

Serum biochemical indexes can reflect the overall health status, physiological stress response, and nutritional status of fish [32,33]. TG and TCHO are the main components of blood lipids [34,35]. The contents of TG and TCHO in serum are important indicators to measure lipid metabolism in fish [36,37]. Studies have shown that α-LA has the effect of lowering blood lipids and could reduce the content of TG and TCHO in mice and rats [38,39,40]. Samuki et al. [12] reported that dietary supplementation of 0.3, 0.6, and 0.9 g/kg α-LA reduced the content of TG in the serum of giant gourami; similarly, Siagian et al. [2] also reported that 1.0 and 1.5 g/kg α-LA reduced the content of TG in the serum of African catfish. In this experiment, α-LA not only reduced the content of TG in L3 serum but also reduced the content of TCHO. Butler et al. [41] suggested that α-LA could reduce the TG content in blood and the liver by inhibiting the expression of liver lipogenic genes, reducing hepatic TG secretion, and stimulating the clearance of TG-rich lipoproteins. Zulkhairi et al. [42] believed that α-LA may reduce the TCHO content in the blood by cholesterol metabolism or lipoprotein lipase activity in the liver. TP and ALB are important indicators of protein synthesis and metabolism and immune function [20,43,44]. Shi et al. [9] discovered that α-LA regulates the AMPKα-CPT-1α pathway to reduce protein consumption in grass carp to increase protein deposition. In addition, Liu et al. [8] found that α-LA could enhance the immune function of the grass carp skin, spleen, and head kidney. In this experiment, the contents of TP and ALB in the serum of L3 were significantly increased, but the growth performance of L3 did not change significantly. Therefore, the increase in TP and ALB may be the result of α-LA enhancing the immune function of juvenile hybrid groupers.

ALT and AST are low in serum and are mainly distributed in liver cells. When liver cells are damaged, they can release ALT and AST to increase their activity in serum, which is consistent with the extent of hepatic cell damage [45]. In this experiment, the levels of ALT and AST in the serum of L3 decreased significantly, which was similar to the experimental results of adding α-LA in grass carp [10] and African catfish [2]. This indicated that the degree of hepatic damage of L3 was lower than that of SL0, i.e., dietary supplementation of α-LA could improve the damage of liver cells in juvenile hybrid groupers. At the same time, by observing the morphology of hepatic tissue cells, it was found that compared with SL0, the morphology of hepatic tissue cells of juvenile hybrid groupers fed with α-LA was improved to varying degrees. This further confirmed that dietary supplementation of α-LA can improve hepatic cell damage.

The antioxidant system can protect fish from oxidative stress and is essential for fish health [46]. Antioxidant enzymes (GSH-Px, CAT, and SOD) can scavenge free radicals to reduce oxidative stress, and their activities can reflect the health status of aquatic animals [47]. GSH-Px can remove hydrogen peroxide and lipid peroxide in the body [48]. SOD is a common antioxidant enzyme in the body and can remove superoxide anions [49]. α-LA is considered to be an “ideal antioxidant” or “general antioxidant”, which can reduce oxidative damage and enhance the antioxidant defense systems of fish by scavenging excessive ROS and regenerating other antioxidants [50,51]. In this experiment, the activity of GSH-Px in the livers of juvenile hybrid groupers fed with α-LA was increased to varying degrees, and the activity of SOD in L2 and L3 was significantly increased. At present, many studies have reported similar results, and α-LA could improve the antioxidant capacity of aquatic animals. Xu et al. [11] found that 0.3 g/kg α-LA significantly increased the activities of SOD and GSH-Px in the liver of Nile tilapia. Li et al. [14] discovered that 600, 900, and 1200 mg/kg α-LA significantly increased the activities of SOD and GSH-Px in the liver, head kidney, and spleen of northern snakehead. Zhang et al. [29] found that 800 mg/kg α-LA significantly increased the activities of SOD and GSH-Px in abalone. In summary, the results of this experiment showed that an appropriate amount of α-LA could increase the activity of antioxidant enzymes in the livers of juvenile hybrid groupers, thereby enhancing antioxidant capacity.

The transcriptome includes RNA transcripts expressed in a specific cell or tissue types under environmental conditions or specific developmental conditions [52]. In recent years, transcriptome analysis has been widely used in aquaculture, and can be used for effective identification and expression analysis of candidate genes, such as growth, development, reproduction, disease, immunity, stress, and toxicology genes [53]. In this experiment, according to serum biochemical indicators and liver antioxidant capacity, liver samples of SL0 and L3 were selected for transcriptome sequencing analysis. Functional analysis of the Kyoto Encyclopedia of Genes and Genomes (KEGG) showed that a total of 10,810 unigenes were annotated into 48 KEGG pathways, of which 12 pathways were significantly enriched, including the JAK/STAT signaling pathway, prolactin signaling pathway, antigen processing and presentation, glycolysis/gluconeogenesis, and so on.

The JAK/STAT signaling pathway is a common pathway for signal transduction of many cytokines, which is closely related to apoptosis, cell proliferation, inflammatory response, and differentiation. It is very important for coordinating adaptive immune mechanisms, initiating innate immunity, and inhibiting inflammatory responses [54]. In this experiment, the JAK/STAT signaling pathway included three genes: *ifnk*, *prl4a1*, and *prl3b1*. In addition, the prolactin signaling pathway also includes *prl4a1* and *prl3b1* genes. The *ifnk* gene is a new type I interferon subclass [55]. Interferons are proteins that are crucial to the human immune system. They are formed in various cells of fish, mammals, reptiles, and amphibians. IFN type I and IFN type II are found in ray-finned fish (Actinopterygii), and IFN type III is also found in phylogenetically older cartilaginous fishes. IFN type I in ray-finned fish (Actinopterygii) can activate the JAK/STAT signaling pathway and induce the expression of IFN-stimulated genes containing IFN-stimulated response elements complexes and, thus, possessing antiviral activity. In addition, in Perciformes, IFN I has been shown to exert antibacterial effects through macrophage phagocytosis [56]. The grouper belongs to Osteichthyes, Actinopterygii, and Perciformes. Both *prl4a1* and *prl3b1* are members of the prolactin family, and prolactin is a multifunctional polypeptide hormone with immunomodulatory and protective effects [57]. Studies have shown that prolactin can induce the expression of genes encoding major phagocytic NADPH oxidase components and ROS production in fish macrophages through the JAK2/Stat/IRF-1 signaling pathway [58]. Antigen processing and presentation is the mechanism by which the entire antigen is degraded and loaded onto MHC molecules (class I and II) to display on the cell surface of T cells [59]. Zhang and Chen [60] found that a novel CC chemokine may be involved in the adaptive immune response by regulating MHC class I antigen processing and presentation in large yellow croaker (*Pseudosciaena crocea*; Richardson, 1846). In this experiment, only the expression of the *ctsl* gene was significantly up-regulated in antigen processing and presentation. Cathepsin L (*ctsl*) is a member of the papain family of cysteine proteases [61] which plays an important role in the biological activities of fish, including antigen processing [62], antigen presentation [63], protein degradation [64], and anti-microbial invasion [65]. Recently, the key role of *ctsl* in the innate immune system of many fish species has been further revealed [66]. In summary, combined with the significant increase in TP and ALB in the serum of L3 and the significant up-regulation in *ifnk*, *prl4a1*, *prl3b1*, and *ctsl* in the JAK/STAT signaling pathway, prolactin signaling pathway, and antigen processing and presentation in liver, it is speculated that dietary supplementation of α-LA can enhance the immune function of juvenile hybrid groupers by regulating the JAK/STAT signaling pathway, prolactin signaling pathway, and antigen processing and presentation.

Glycolysis/gluconeogenesis is an opposing metabolic pathway involved in carbohydrate degradation and synthesis and plays an important role in maintaining glucose homeostasis [67]. In this experiment, the glycolysis/gluconeogenesis pathway included two genes, *gapdh* and *eno1*. Glyceraldehyde-3-phosphate dehydrogenase (*gapdh*) plays a key role in the glycolytic pathway. It can catalyze the formation of glyceraldehyde-3-phosphate to 1,3-bisphosphoglycerate, which produces NADH. NADH can synthesize ATP through the electron transport chain in mitochondria [68]. α-enolase (*eno1*) plays a functional role in glycolysis/gluconeogenesis. It can catalyze the conversion of 2-phosphate-D-glycerate to phosphoenolpyruvic acid during glycolysis and phosphoenolpyruvic acid to 2-phosphate-D-glycerate during glycogen synthesis [69]. Huang et al. [15] showed that the addition of α-LA to the diet could enhance the expression of glycolysis, gluconeogenesis, and glucose transport-related genes in zebrafish (*Danio rerio*; Hamilton, 1822) livers. In this experiment, the expression of *gapdh* gene was significantly down-regulated, and the expression of eno1 gene was significantly up-regulated. Therefore, it is speculated that α-LA can maintain the glucose homeostasis of juvenile hybrid groupers by regulating the expression of *gapdh* and *eno1* genes in the glycolysis/gluconeogenesis pathway.

Therefore, the optimal addition of α-LA in the diet of hybrid groupers needs further study. However, this experiment showed that without affecting the growth of hybrid groupers, it could reduce the blood lipid level of hybrid groupers, improve the damage of liver cells, and increase the activity of antioxidant enzymes in the liver. This indicates that an appropriate amount of α-LA can be used as an additive to improve fish health in actual production. In addition, the transcriptome results provide some theoretical knowledge for the further study of α-LA in immune and glucose homeostasis.

## 5. Conclusions

In summary, in this experiment, 0.4 and 0.6 g/kg α-LA inhibited the growth performance of juvenile hybrid groupers. Although 1.2 g/kg α-LA had no significant effect on the growth performance, it could reduce the blood lipid level of juvenile hybrid groupers, improve hepatocyte damage, and increase the antioxidant enzyme activity of the liver. Transcriptome analysis showed that dietary α-LA supplementation significantly affected the pathway related to immune function (JAK/STAT signaling pathway, prolactin signaling pathway, and antigen processing and presentation), and significantly up-regulated the expression of genes related to immunity (*ifnk*, *prl4a1*, *prl3b1*, and *ctsl*). In addition, α-LA also changed the pathway related to glucose homeostasis (glycolysis/gluconeogenesis), significantly down-regulated the expression of the *gapdh* gene, and up-regulated the expression of the *eno1* gene.

## Figures and Tables

**Table 1:** Composition and nutritional levels of the experimental diets (g/kg).

Ingredients	Diet Groups			
	SL0	L1	L2	L3
Fish meal	420	420	420	420
Shrimp med	50	50	50	50
Spray-dried blood meal	20	20	20	20
Soybean meal	160	160	160	160
Wheat gluten	50	50	50	50
Cottonseed meal	50	50	50	50
Wheat flour	150	149.6	149.4	148.8
Soybean lecithin	30	30	30	30
Fish oil	40	40	40	40
Alpha lipoic acid	0.0	0.4	0.6	1.2
Calcium monophosphate	10	10	10	10
Vitamin and mineral premix ^1^	20	20	20	20
Total	1000.0	1000.0	1000.0	1000.0
Nutrient levels				
Moisture	9.2	9.5	10.0	9.1
Crude protein	49.6	49.3	49.4	49.7
Crude lipid	10.4	10.3	10.3	10.3
Ash	11.0	10.9	10.8	11.0

^1^ Vitamin and mineral premix (g/kg of mixture): vitamin A, 5.0 g; vitamin B_1_, 10.0 g; vitamin B_2_, 10.25 g; vitamin B_6_, 22.5 g; vitamin B_12_, 0.05 g; vitamin D, 20.0 g; vitamin E, 45.0 g; vitamin K_3_, 2.275 g; Ca(H_2_PO_4_)_2_·H_2_O, 25.0 g; CoCl_2_·6H_2_O, 2.175 g; CuSO_4_·5H_2_O, 9.425 g; KIO_3_, 0.015 g; KCl, 8.87 g; Na_2_SeO_3_, 1.0 g; MgSO_4_·H_2_O, 6.095 g; MnSO_4_·7H_2_O, 0.055 g; ZnSO_4_·7H_2_O, 14.56 g; biotin, 1.0 g; cellulose, 239.485 g; D-calcium pantothenate, 30.335 g; folic acid, 3.085 g; ferric citrate, 7.175 g; inositol, 70.02 g; nicotinic acid, 41.0 g; zeolite powder, 425.63 g.

**Table 2:** Primer sequences for RT-qPCR verification.

Gene Name	Primer Sequence	Accession Number	Product Length
*β-actin*	F: GCAGGAGTACGATGAGTCCG	KU200949.2	102
	R: GCTGAAGTTGTTGGGCGTTT		
*i-1*	F: TCCCCAACCATAACGCTGAAC	Unigene0003248	115
	R: CAGACCACTGTTTTCTCATCTCCGT		
*eno1*	F: CATCAACGGCGGCTCACAT	Unigene0018779	185
	R: CGAAACCTCCCTCGTCTCCTACA		
*txnip*	F: CGGCAGGTCCTTCTACAGCA	Unigene0047552	146
	R: GGCTCTTGAGTTTCTTCCCACC		
*pvalb*	F: TGGTTTCATTGAGGAGGAAGAGC	Unigene0012447	128
	R: CAATCTTGCCATCACCGTCAG		
*dusp1*	F: CGTCCGCTTCAGCACCATA	Unigene0043927	155
	R: TCGCCTGGCTCAAGTCAAAG		

**Table 3:** Effect of diet supplementation with α-LA on the growth performance, morphology, and feed utilization of juvenile hybrid groupers.

Parameter	Diet Groups			
	SL0	L1	L2	L3
IBW (g)	23.88 ± 0.30	23.98 ± 0.23	24.13 ± 0.06	24.10 ± 0.00
FBW (g)	103.42 ± 6.13	98.87 ± 8.30	95.65 ± 4.38	105.60 ± 4.72
WGR (%)	333.26 ± 30.76 ^b^	312.25 ± 3.37 ^a^	296.33 ± 17.75 ^a^	338.19 ± 19.59 ^b^
SR (%)	100.00 ± 0.00	100.00 ± 0.00	100.00 ± 0.00	96.67 ± 2.89
FCR	0.82 ± 0.06 ^a^	0.83 ± 0.05 ^a^	0.88 ± 0.06 ^b^	0.81 ± 0.04 ^a^
SGR (%/d)	2.62 ± 0.13	2.53 ± 0.01	2.46 ± 0.08	2.64 ± 0.08
CF (g/cm^3^)	6.56 ± 0.69	6.52 ± 0.82	7.07 ± 0.46	6.96 ± 0.71
FI (%)	1.59 ± 0.07	1.60 ± 0.11	1.67 ± 0.08	1.58 ± 0.04
VSI (%)	12.33 ± 1.12	13.0 ± 1.41	12.44 ± 2.07	12.11 ± 1.27
HSI (%)	3.30 ± 0.70	3.38 ± 0.79	3.54 ± 0.82	3.46 ± 0.75

Notes: data are expressed as mean ± SD (n  =  3), and values with different superscript letters in the same row are statistically significant (*p* < 0.05). IBW, initial body weight; FBW, final body weight; WGR, weight gain rate; SR, survival rate; FCR, feed conversion ratio; SGR, specific growth rate; CF, condition factor; FI, feed intake; VSI, viscerosomatic index; HIS, hepatosomatic index.

**Table 4:** Effect of diet supplementation with α-LA on the serum biochemical indexes of juvenile hybrid groupers.

Parameter	Diet Groups			
	SL0	L1	L2	L3
TG (mmol/L)	1.19 ± 0.22 ^b^	1.05 ± 0.11 ^b^	1.01 ± 0.05 ^ab^	0.80 ± 0.06 ^a^
TCHO (mmol/L)	1.60 ± 0.14 ^b^	1.40 ± 0.07 ^ab^	1.40 ± 0.06 ^ab^	1.21 ± 0.15 ^a^
TP (g/L)	19.32 ± 0.58 ^a^	24.42 ± 1.57 ^b^	28.48 ± 2.85 ^bc^	29.44 ± 3.52 ^c^
ALB (g/L)	4.45 ± 0.32 ^ab^	4.03 ± 0.51 ^a^	5.26 ± 0.70 ^bc^	5.99 ± 0.35 ^c^
LDL-C (mmol/L)	0.34 ± 0.05 ^a^	0.29 ± 0.01 ^a^	0.80 ± 0.02 ^c^	0.42 ± 0.06 ^b^
HDL-C (mmol/L)	1.10 ± 0.12	1.25 ± 0.06	1.11 ± 0.18	1.37 ± 0.26
AST (U/L)	41.89 ± 3.77 ^b^	43.78 ± 4.38 ^b^	44.23 ± 2.42 ^b^	33.40 ± 2.12 ^a^
ALT (U/L)	581.11 ± 18.34 ^b^	400.42 ± 45.54 ^a^	382.46 ± 30.51 ^a^	390.53 ± 19.07 ^a^

Notes: data are expressed as mean ± SD (n  =  3), and values with different superscript letters in the same row are statistically significant (*p* < 0.05). TG, triglyceride; TCHO, total cholesterol; TP, total protein; ALB, albumin; LDL-C, low-density lipoprotein cholesterol; HDL-C, high-density lipoprotein cholesterol; AST, aspartate aminotransferase; ALT, alanine aminotransferase.

**Table 5:** Effect of diet supplementation with α-LA on the antioxidant capacity of the livers of juvenile hybrid groupers.

Parameter	Diet Groups			
	SL0	L1	L2	L3
GSH-Px (U/mgprot)	312.35 ± 12.26 ^a^	346.87 ± 6.48 ^b^	350.37 ± 6.88 ^b^	371.72 ± 7.18 ^c^
CAT (U/mgprot)	10.41 ± 0.36	11.28 ± 0.78	12.59 ± 1.40	10.47 ± 0.42
SOD (U/mgprot)	51.64 ± 1.61 ^a^	44.02 ± 3.86 ^a^	62.01 ± 4.33 ^b^	61.03 ± 5.33 ^b^
MDA (nmol/mgprot)	1.60 ± 0.43	1.24 ± 0.15	1.30 ± 0.06	1.22 ± 0.03

Notes: data are expressed as mean ± SD (n  =  3), and values with different superscript letters in the same row are statistically significant (*p* < 0.05). GSH-Px, glutathione peroxidase; CAT, catalase; SOD, superoxide dismutase; MDA, malondialdehyde.

**Table 6:** Information of the transcriptomic read of each sample.

Sample	RawData (bp)	CleanData (bp)	Q20 (%)	Q30 (%)	GC (%)
SL0-1	7,981,551,300	7,908,021,279	98.09%	94.62%	49.83%
SL0-2	5,738,949,600	5,643,980,098	98.15%	94.73%	50.02%
SL0-3	5,812,150,800	5,713,500,475	98.19%	94.82%	49.65%
L3-1	6,519,141,900	6,430,009,609	98.13%	94.69%	50.10%
L3-2	5,679,499,500	5,590,980,794	98.22%	94.86%	50.08%
L3-3	6,029,491,800	5,943,136,963	98.13%	94.66%	49.58%

**Table 7:** Part of the DEGs of SL0 vs L3.

Genes ID	Genes	Description	log2(FC)	*p*-Value	FDR
Unigene0038423	*ifnk*	Interferon kappa precursor	+10.632	0.000	0.039
Unigene0049822	*prl3b1*	Prolactin-3B1 precursor	+12.004	0.000	0.000
Unigene0036965	*prl4a1*	Prolactin-4A1 precursor	+11.728	0.000	0.000
Unigene0046207	*ctsl*	Cathepsin R precursor	+7.349	0.000	0.000
Unigene0018198	*gapdh*	Glyceraldehyde-3-phosphate dehydrogenase	−6.806	0.000	0.000
Unigene0018779	*eno1*	enolase	+1.640	0.000	0.010
Unigene0003248	*i-1*	Beta-galactoside-binding lectin-like isoform X1	+2.216	0.000	0.001
Unigene0012447	*pvalb*	Parvalbumin beta-like	−7.204	0.000	0.000
Unigene0043927	*dusp1*	MAP kinase phosphatase 1	−1.880	0.000	0.015
Unigene0047552	*txnip*	Thioredoxin-interacting protein-like	+1.577	0.000	0.034

Notes: “+” and “-” represent up-regulation and down-regulation of the expression of DEDs, respectively.

## Data Availability

Upon reasonable request, the data supporting this study are accessible from the corresponding author.
